# Development of a Patient-Derived Xenograft (PDX) of Breast Cancer Bone Metastasis in a Zebrafish Model

**DOI:** 10.3390/ijms17081375

**Published:** 2016-08-22

**Authors:** Laura Mercatali, Federico La Manna, Arwin Groenewoud, Roberto Casadei, Federica Recine, Giacomo Miserocchi, Federica Pieri, Chiara Liverani, Alberto Bongiovanni, Chiara Spadazzi, Alessandro de Vita, Gabri van der Pluijm, Andrea Giorgini, Roberto Biagini, Dino Amadori, Toni Ibrahim, Ewa Snaar-Jagalska

**Affiliations:** 1 Osteoncology and Rare Tumors Center, Istituto Scientifico Romagnolo per lo Studio e la Cura dei Tumori (IRST) IRCCS, Via P. Maroncelli 40, 47014 Meldola, Italy; federico.lamanna@gmail.com (F.L.M.); federica.recine@irst.emr.it (F.R.); giacomo.miserocchi@irst.emr.it (G.M.); chiara.liverani@irst.emr.it (C.L.); alberto.bongiovanni@irst.emr.it (A.B.); chiara.spadazzi@irst.emr.it (C.S.); alessandro.devita@irst.emr.it (A.d.V.); dino.amadori@irst.emr.it (D.A.); toni.ibrahim@irst.emr.it (T.I.); 2 Department of Urology, Leiden University Medical Center, J-3-100, Albinusdreef 2, 2333ZA Leiden, The Netherlands; g.van.der.pluijm@umail.leidenuniv.nl; 3 Department of Molecular Cell Biology, Institute of Biology, Leiden University, Sylviusweg 72, 2333BE Leiden, The Netherlands; a.groenewoud@biology.leidenuniv.nl (A.G.); b.e.snaar-jagalska@biology.leidenuniv.nl (E.S.-J.); 4 Department of Orthopedics, Istituto Ortopedico Rizzoli, University of Bologna, Via Pupilli, 1, 40136 Bologna, Italy; roberto.casadei@ior.it; 5 Pathology Unit, Morgagni-Pierantoni Hospital, 47121 Forlì, Italy; federica.pieri@auslromagna.it; 6 Department of Medical and Surgical Sciences for Children and Adults, Modena Polyclinic, Viale del Pozzo, 71, 41124 Modena, Italy; andreagiorgini@hotmail.it; 7 UOC Orthopedic Surgery, Regina Elena National Cancer Institute, 00144 Rome, Italy; biagini@ifo.it

**Keywords:** bone metastasis, zebrafish model, breast cancer, patient-derived xenograft

## Abstract

Bone metastasis is a complex process that needs to be better understood in order to help clinicians prevent and treat it. Xenografts using patient-derived material (PDX) rather than cancer cell lines are a novel approach that guarantees more clinically realistic results. A primary culture of bone metastasis derived from a 67-year-old patient with breast cancer was cultured and then injected into zebrafish (ZF) embryos to study its metastatic potential. In vivo behavior and results of gene expression analyses of the primary culture were compared with those of cancer cell lines with different metastatic potential (MCF7 and MDA-MB-231). The MCF7 cell line, which has the same hormonal receptor status as the bone metastasis primary culture, did not survive in the in vivo model. Conversely, MDA-MB-231 disseminated and colonized different parts of the ZF, including caudal hematopoietic tissues (CHT), revealing a migratory phenotype. Primary culture cells disseminated and in later stages extravasated from the vessels, engrafting into ZF tissues and reaching the CHT. Primary cell behavior reflected the clinical course of the patient’s medical history. Our results underline the potential for using PDX models in bone metastasis research and outline new methods for the clinical application of this in vivo model.

## 1. Introduction

Metastasis from solid tumors is the main cause of cancer-related death [1]. It is a dynamic, complex and multistep process involving tumor cell intravasation, spread to different organs, extravasation and cancer cell colonization, and final outgrowth of secondary lesions [2]. Bone is the most frequent site of cancer relapse after lung and liver and is one of the most common complications in several primary tumors. In particular, 70% of patients with breast or prostate cancer relapse to bone during the course of their disease [3,4,5,6]. The disease is usually not curable, and about 80% of patients die within five years of the diagnosis of bone metastasis [3].

It was discovered in the late 19th century [7] that primary tumors have distinctive sites of metastasis. Cancer cells that reach bone tissue may stop there and are capable of developing into a secondary lesion through a process known as osteomimicry [1,8], i.e., cells acquire specific features characteristic of bone resident cells which enable them to survive and proliferate in the new microenvironment. For example, cancer cells, such as hematopoietic stem cells (HSCs), are capable of expressing C-X-C motif chemokine receptor 4 (CXCR4) and thus take advantage of the same physiological mechanism of chemoattraction used by HSCs. Cells expressing the same pattern of bone cell integrins may remain in the blood endothelium and escape to bone parenchyma. When breast cancer cells finally arrive in bone tissue, the balance between bone resorption and bone formation is lost, usually in favor of bone resorption, with the consequent development of lytic or mixed lesions. Osteoclast activity causes the release of growth factors from the bone matrix such as transforming growth factor β (TGFβ), which contributes to cancer cell proliferation. Moreover, cancer cells in the bone microenvironment are stimulated to produce cytokines and to secrete factors such as interleukin 6 (IL6) and parathyroid hormone-related protein (PThrP), the latter inducing osteoblasts to increase receptor activator of nuclear factor κB ligand (RANKL) and decrease osteoprogesterin (OPG) production. The change of the RANKL/OPG ratio is one of the major causes of osteoclast differentiation and activity. Briefly, these described relations constitute the backbone of a more complex network of interactions between bone and cancer cells occurring in the bone after their arrival and which are described as the “vicious cycle of bone metastases” [8].

The outstanding scientific findings of recent years have upgraded the role of the bone as one of the most frequent sites of metastasis to that of a key actor and director in the different phases of the entire metastatic process. Cancer cells induce the migration of vascular endothelial growth factor receptor 1 (VEGFR1)-positive bone marrow-derived hematopoietic cells to sites of future metastasis where they form cellular clusters preceding tumor cell arrival and increase fibronectin production and matrix stiffness [9], creating a pre-metastatic niche in tissue [8]. It is worthy of note that cancer cells can remain quiescent in bone marrow for several years until some stimuli determine their reactivation with consequent spreading and formation of a secondary lesion to the bone, to other organs, or to the site of the primary tumor [8,10].

One of the most widely used in vivo models to study cancer cell metastasis is that of xenografts in immunodeficient mice. However, this model is hampered by being time-consuming and labor-intensive. Zebrafish (*Danio rerio*) (ZF), a small freshwater tropical fish, and their transparent embryos recently emerged as a promising xenograft tumor model [11,12,13]. ZF display distinct features that facilitate the exploration of tumor development, angiogenesis, invasion and metastasis. Moreover as a vertebrate, the ZF model shows high levels of physiologic and genetic similarities to mammals, closely mimicking the clinical setting and permitting the natural history of the tumor to be monitored. It is thus thought to be a potentially powerful model for preclinical studies on bone metastases [13].

The majority of preclinical studies on bone metastases use commercially available immortalized cancer cell lines or cancer cell lines selected in vivo to form metastases in bone tissue [14,15,16,17]. However, cell lines, after being cultured in plastic supports and going through hundreds of freeze/thaw passages, modify their phenotypic behavior in order to survive in the new microenvironment, which can seriously compromise results. Thus, the use of primary cultures obtained from tissue resected during surgery could be the answer to overcoming these problems [13,18,19].

The present work focuses on the development of a patient-derived xenograft of a bone metastasis in ZF embryos to study cell behavior during the metastatic process.

## 2. Results

### 2.1. Patient History

A 67-year-old female with breast cancer previously treated with surgery and systemic therapies presented at our institute with bone and liver metastases. The patient was diagnosed with breast cancer in 1999 and a left radical mastectomy was performed, revealing invasive ductal carcinoma G2, pT1c (m), pN0, M0. ER 80% PgR 10%, Ki-67 20% and c-erbB2 0%. Adjuvant hormone therapy with tamoxifen was administered. About 15 years later, the patient developed pain in her left arm and an X-ray revealed the presence of an osteolytic lesion of about 60 × 33 mm in the humerus, suggestive of a secondary bone lesion. Magnetic resonance imaging (MRI) of the left shoulder confirmed the presence of the bone lesion, together with an intra-articular fracture. A total body computed tomography (CT) scan also revealed the presence of a liver lesion 67 × 62 mm in the fifth and sixth hepatic segments. The α-fetoprotein value was normal. A total body bone scan showed a single area of pathological uptake in the left humerus. Biopsy of the bone lesion confirmed the diagnosis of metastasis from ductal carcinoma of the breast with mucinous aspects, estrogen receptor (ER) 100% progesteron receptor (PgR) 70%, Ki-67 15% and c-erbB2 0%.

**Figure 1:**
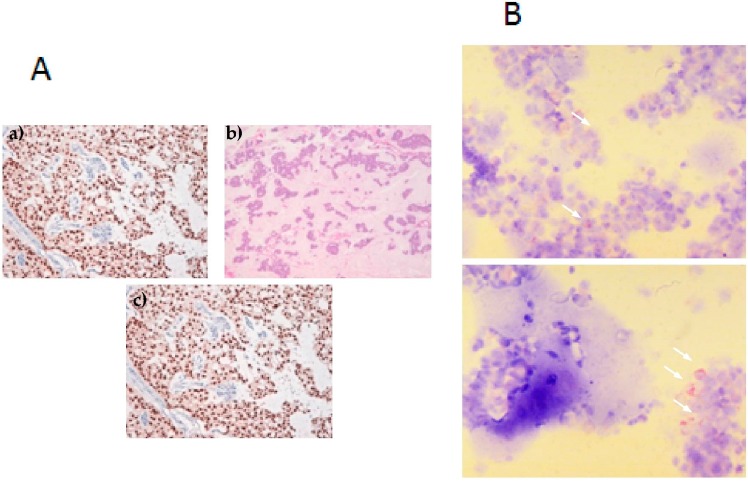
Breast cancer bone metastasis. (**A**): (**a**) Histological hematoxylin and eosin (H & E) staining of bone metastasis (BM) primary culture (5× magnification). Mucinous areas show low cellularity; (**b**) H & E staining of BM primary culture (20× magnification). Monomorphic cells with round nucleus and nucleolus are seen in nests with a minor mucus quantity; (**c**) ER staining showing positivity; (**B**): Cytospin of BM primary cells. White arrows show pancytokeratin-positive cells.

The multidisciplinary medical team examined the case and recommended surgery and hormone treatment with anastrozole and bone-targeted therapy with zoledronic acid. The patient underwent resection and prosthetic replacement of the humerus. Histology confirmed metastasis compatible with breast cancer, ER 100% PgR 5%, Ki-67 5% and c-erbB2 0%. Surgical margins were negative (Figure 1). The hepatic lesion was removed, histological analysis confirming metastasis of breast cancer origin (ER 100%, Ki-67 20%, c-erbB2 0%).

There was no evidence of macroscopic disease at the most recent radiological evaluation (CT scan). Treatment with anastrozole and zoledronic acid is ongoing and is well tolerated by the patient.

### 2.2. Breast Cancer Cell Lines

**Figure 2:**
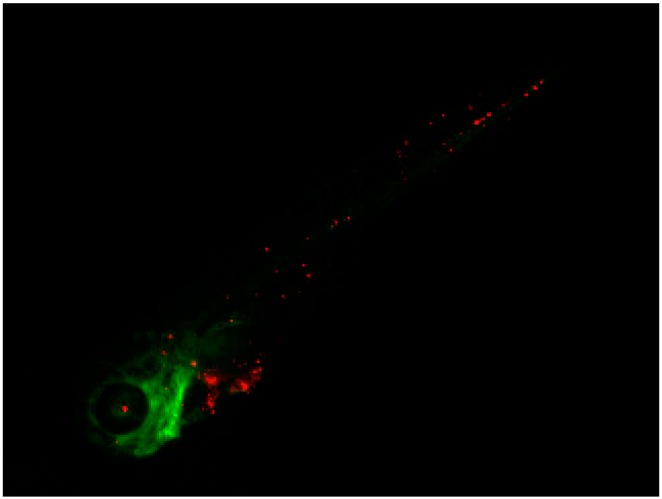
Representative image of MDA-MB-231 cell line five days after injection into the duct of Couvier of 2 day post fertilization (dpf), *Tg*(*fli1*:*GFP*) ZF embryo. MDA-MB-231, labeled in red, were monitored on a daily basis for the duration of the experiment (five days) and showed progressive and extensive dissemination throughout the developing embryo (25× magnification).

The in vivo behavior of cells from a bone metastasis (BM) primary culture was compared with that of two different breast cancer cell lines: MCF7, a non-invasive, hormone receptor–positive line (like the BM primary culture), and MDA-MB 231, a highly invasive, triple-negative breast cancer line widely used for in vivo studies on breast cancer metastasis. MCF7 cells did not develop a phenotype in the in vivo model and none survived the five-day duration of the experiment. Conversely, MDA-MB-231 disseminated and colonized different parts of the zebrafish (ZF), including caudal hematopoietic tissue (CHT), indicating a migratory phenotype (Figure 2).

### 2.3. Invasive Phenotype of Primary Culture of Bone Metastasis in Zebrafish Model

**Figure 3:**
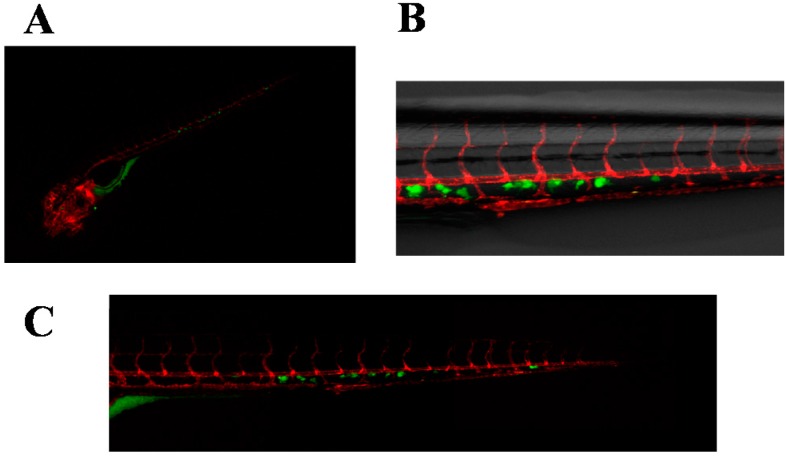
Images of carboxyfluorescein succinimidyl ester (CFSE)-labeled, patient-derived breast cancer bone metastasis (primary cells) xenografted in *Tg*(*kdrl*:mcherry) ZF embryos. (**A**) Whole-body image of the zebrafish embryo at 5 dpi (25× magnification). Primary cells disseminated predominantly in the caudal hematopoietic tissues (CHT) of the embryo; (**B**) Details of (**A**), showing the interactions of primary cells with the zebrafish vessels in the CHT. Cells extravasated in the CHT of the ZF embryo and engrafted in close proximity of the vessels. Fluorescent images merged with brightfield image, 63× magnification; (**C**) Combined picture of the CHT of embryo in (**A**,**B**). Individual images were taken at 63× magnification.

Positivity to pan-cytokeratin staining confirmed the presence of cancer cells in the primary culture. In particular, a mean of 12% of cells per field was observed (20× magnification). Cells derived from the primary culture and selected by colony picking, as reported in the Experimental Section, were injected into the duct of Cuvier of 2 dpf *Tg*(*kdrl*:*mCherry*) ZF. The zebrafish model was used primarily to stabilize the surgical material with an in vivo near-patient model and to assess the invasiveness of the selected cancer cell population. It also enabled us to track migratory movements of injected cells across the tissues of the developing zebrafish embryos at the single-cell level. Injected cells survived and disseminated in the ZF embryos, extravasating and engrafting mainly in the perivascular milieu of the CHT (Figure 3). The cells remained visible for the entire duration of the in vivo experiment.

### 2.4. Overexpression of Osteomimicry Markers by Primary Culture of Bone Metastasis

**Figure 4:**
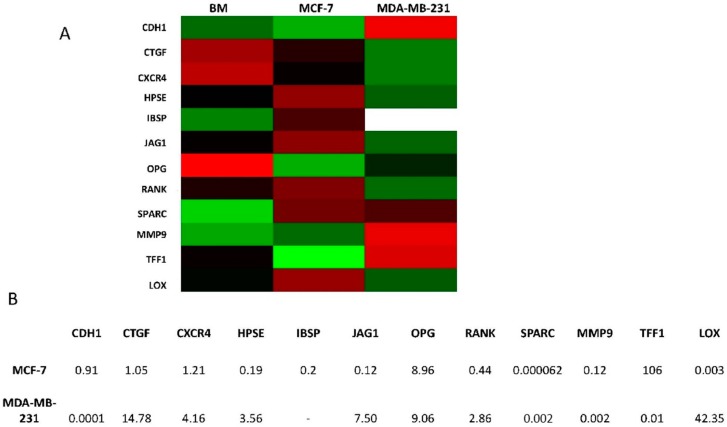
Gene expression analysis of aggressiveness and osteomimicry markers. (**A**) Heatmap configuration of gene expression analysis in BM primary culture and breast cancer cell lines; green/red bars refer to high/low gene expression, respectively; black bars refer to intermediate levels of expression; (**B**) Gene expression quantification by comparative threshold cycle (*C*_t_) value method (∆∆*C*_t_). The primary culture was chosen as calibrator. See the Experimental Section for selected genes.

Several markers involved in breast cancer cell aggressiveness and osteomimicry were evaluated in the BM primary culture and in the MCF7 and MDA-MB-231 cell lines. Secreted protein acidic and rich in cysteine (SPARC) expression in the primary culture was almost 16,000-fold higher than that of MCF7 cell lines and 2000-fold higher than that of MDA-MB-231. Integrin-binding sialoprotein (IBSP), another osteomimicry marker, and matrix metalloprotease 9 (MMP9), a marker of cancer cell aggressiveness expressed by osteoclasts in bone tissue, were also overexpressed in the BM primary culture. Furthermore, higher levels of heparanase (HPSE), JAGGED1 (JAG1) and receptor activator of nuclear factor κB (RANK) were observed in BM cells than in MCF7, a cell line with a hormone receptor arrangement similar to that of the primary culture (Figure 4).

## 3. Discussion

Although the prognosis of patients with bone metastases from breast cancer is poor, in recent years bone-targeted therapies used in combination with anticancer agents have substantially improved quality of life and survival [8,20].

Numerous studies have used immunodeficient mice to develop human-to-mouse xenograft tumor models to study the metastasis process and identify key steps and genes involved so that novel markers and new targets for the development of innovative drugs can be derived [8,14,16].

Current experimental procedures have limitations because of the length of time it takes for tumors to grow and spread, the variation in the tumor growth rate in vivo, and the high costs of breeding and housing large numbers of mice. The ZF model has a number of important advantages over traditional mouse models, i.e., simplicity of genetic manipulation, inexpensive housing, rapid embryonic development, and easy visualization of internal structures. Furthermore, as fish produce 100–200 eggs/mating, in vivo experiments are designed with a higher number of replicates than those of mice models, leading to a higher statistical power and opening up new possibilities for detecting rare phenotypes. Some studies have shown that human tumor cells proliferate and interact with vessel tissues in ZF embryos without the risk of rejection as the latter are devoid of a mature immune system [21,22,23,24]. ZF models have thus become a valid alternative for overcoming mice model limitations, e.g., when a high number of cells need to be injected. The majority of studies on the metastasis process take advantage of commercially available immortalized cancer cell lines [14,15,16,17] which, in adapting to their new environment, tend to lose cell heterogeneity and compensate for the loss of stromal contribution. During the process of adaptation, clones with a higher proliferative rate than that of the primary tumor and thus not representative of the cancer cell population may be selected [13]. Surgical material would perhaps be more suitable to develop patient-derived xenografts in which the stromal counterpart and cancer cell heterogeneity are both preserved.

However, as the quantity of material available is often insufficient for use in murine models, the ZF becomes the perfect candidates to substitute mice models. As such, 100–200 replicates can be performed with as few as 400–500,000 cells, providing high reproducibility and robust results. Some researchers have developed PDXs in ZF models to study human cancer cell behavior, including response to therapy [18,19,25,26]. The ZF has also shown to be a suitable model to study metastases as cancer cells with different in vitro invasive potential preserve this behavior after injection into ZF embryos [26].

The current study focused on the development of a PDX from a breast cancer bone metastasis in ZF embryos. We first validated this in vivo model by testing the ability of two well-established breast cancer cell lines to colonize the CHT of two-day post-fertilization (dpf) ZF embryos. We used a red dye, CM-DiI dye, commonly used in this model, to label the cell lines. One disadvantage of this dye is that, after injection into the ZF embryos, labeled cells frequently produce debris and apoptotic bodies at later time points, lowering the signal-to-noise ratio and thus hampering the correct quantification of the phenotype. We therefore set up a labeling strategy based on the green dye carboxyfluorescein succinimidyl ester (CFSE) to obtain a clearer phenotype for the injection of the primary bone metastasis culture.

The hormone receptor–positive MCF7 cell line did not colonize the CHT efficiently, whereas the highly aggressive and triple-negative cell line MDA-MB-231 showed efficient and widespread colonization of the ZF embryo including the CHT. A comparison between the grafting potential of the patient-derived bone metastasis and that of the two breast cancer cell lines enabled us to evaluate whether the detected phenotype of the patient-derived cells could be ascribed to the common tissue of origin of the cells. Despite the histological and molecular similarities, i.e., tissue of origin and hormone receptor pattern of MCF7 cells, respectively, the patient-derived cells showed a specific grafting pattern with cells mainly colonizing the CHT of the ZF, suggesting that the in vivo behavior of these cells may be due to an adaptation process to the bone tissue of the patient. As the CHT is the first homing and expansion site for hematopoietic stem cells in the ZF embryo [27], the experimental phenotype can be considered as an expression of bone marrow tropism [28]. A more detailed and mechanistic description of the process of dissemination and colonization of the CHT by cancer cells can be found in the study by He et al. [29].

This technique produced an aspecific signal detectable in the intestinal tract of the zebrafish due to dye leakage at early time points, but provided a crisp fluorescent signal at later time points.

Although the hormone receptor–positive MCF7 cell line did not efficiently colonize the CHT, the highly aggressive and triple-negative cell line MDA-MB-231 showed efficient widespread CHT colonization and local growth. We then assayed cells collected from a lytic bone lesion of a patient with metastatic hormone receptor–positive breast cancer and showed, for the first time in this model, engraftment and local growth of the ex vivo cells. Furthermore, cells colonized different parts of the embryos, showing an aggressive phenotype. As the CHT is the primary hematopoietic organ of the zebrafish, the experimental phenotype can be considered as an expression of bone tropism. Although the primary culture had the same hormonal receptor pattern as that of MCF7, its behavior differed substantially as MCF7 cells did not show a phenotype in vivo, as previously reported. The phenotype of the primary culture resembled that of the invasive cell line MDA-MB-231. Our in vivo results appear to reflect the patient’s clinical history; cells were initially quiescent and then re-activated, going on to develop concurrent bone and hepatic lesions. Fifteen years after the primary diagnosis of breast cancer, the patient developed bone and hepatic disease, both as single metastatic localizations. The presence of visceral disease is suggestive of a different and more aggressive phenotype than that of the indolent primary tumor.

We also studied the gene expression profile of the breast cancer cell lines and the primary culture for markers of osteomimicry and aggressiveness. As the primary cell population was heterogeneous, its gene expression profile was not ascribable to either of the two cancer cell lines. Of note, SPARC expression was higher in the BM primary culture than in MCF7 and MDA-MB-231. This protein, also known as osteonectin, is normally expressed by resident bone cells and overexpressed by cancer cells in osteomimicry [30]. It is also thought to play a role in the chemoattraction of both breast and prostate cancer cells [31] and has been proposed as a biomarker for bone metastasis [32]. As SPARC is expressed by normal bone cells, it was no surprise to find high levels of it in the bone metastasis primary culture. The primary culture also overexpressed other markers (Jagged1 and RANK) [33,34,35,36].

## 4. Experimental Section

### 4.1. Cell Cultures

The experiments were performed on MDA-MB-231, a triple-negative human breast cancer cell line, and on MCF7, a hormone receptor–positive breast cancer cell line, both obtained from the America Type Culture Collection (Rockville, MD, USA). Cells were cultured as a monolayer in 75-cm^2^ flasks at 37 °C in Dulbecco’s Modified Eagle’s medium (DMEM) medium (PAA, Piscataway, NJ, USA) supplemented with 10% fetal bovine serum and 1% glutamine (PAA) and 10% penicillin/streptomycin in a 5% CO_2_ atmosphere. Cells were cultured until they reached 90%–100% confluence and then supplemented with fresh DMEM medium, which was collected after 24 h, filtered through a 0.22 μm filter, aliquoted and stored at −20 °C.

### 4.2. Isolation of Primary Bone Metastasis Cells

The bone tumor specimen was obtained from a bone metastasis resected from the left humerus of a 67-year-old woman with a previous history of breast cancer. The protocol IRST B039 was reviewed and approved by the ethics Committee IRST IRCCS AVR and by the Medical Scientific Committee of IRST in February 2015. The study was performed in accordance with the principles of Good Clinical Practice and the Helsinki Declaration. The patient gave written informed consent to take part in the study.

The surgical material was analyzed and selected by an experienced pathologist and processed within 3 h of removal. The specimen was washed twice in sterile phosphate buffered saline (PBS) supplemented with 10% penicillin/streptomycin and disaggregated into 1–2 mm^3^ pieces with sterile surgical blades. The obtained fragments were incubated with 2 mg/mL collagenase type I (Millipore Corporation, Billerica, MA, USA) at 37 °C under stirring conditions. The enzymatic digestion was stopped after 2 h by adding complete DMEM medium. The cell suspension was then filtered with 100 µm sterile Filters (CellTrics, Partec, Münster, Germany). Cells were counted and cultured at a density of 80,000 cells/cm^2^ in standard monolayer cultures. All cells were maintained in complete DMEM medium at 37 °C in a 5% CO_2_ atmosphere.

After a seven-day culture, a colony of cells that morphologically resembled cancer cells was selected with a plastic ring (Sigma, St. Luois, MO, USA), trypsinized and reseeded in a different well. The obtained cell population was expanded through a further three to four passages so that it could be used for subsequent experiments.

### 4.3. Immunocytochemistry

The primary culture suspension was counted and cytocentrifuged for 8 min at 900 rpm onto glass slides at a concentration of 2 × 10^5^ cells/spot. The cytospin preparations were fixed in acetone and chloroform, air-dried overnight and stored at −20 °C. Immunostaining to detect CK-positive cells was performed with the Epimet^®^ kit (Micromet, Deutschland GmbH, Düsseldorf, Germany) which uses the monoclonal antibody A45-B/B3, a pancytokeratin marker.

### 4.4. Prepapration of Cells for Implantation into Zebrafish (ZF) Embryos

MDA-MB-231 and MCF7 cell lines and the primary culture were gently washed with PBS and harvested with trypsin, counted and resuspended at a density of 10^6^/mL. The breast cancer cell lines were stained with CMDiI dye (Sigma), while the primary culture was stained with CellTrace™ CFSE Cell Proliferation Kit (Life Technologies, Carlsbad, CA, USA), in accordance with the manufacturers’ instructions. Each labeled cell suspension was loaded into borosilicate glass capillary needles (1 mm outer diameter × 0.78 mm inner diameter; Harvard Apparatus, Saint-Laurent, QC, Canada) and injected within 3 h of cell harvest.

### 4.5. In Vivo Experiments with the ZF Model

The ZF transgenic lines *Tg(kdrl:mCherry)* and *Tg(fli1:GFP)* were used for the in vivo studies of the breast cancer cell lines and primary culture, respectively. Zebrafish and embryos were raised, staged and maintained according to standard procedures in compliance with the local animal welfare regulations and the EU Animal Protection Directive 2010/63/EU. *N*-phenylthiourea 0.2 mM (Sigma) was applied to prevent pigment formation from the first dpf.

Two-dpf ZF embryos were anesthetized with 0.003% tricaine (Sigma) and positioned on a 10 cm petri dish coated with 1% agarose. Then 50–400 manually counted cells were injected into the duct of Cuvier using a Pneumatic Pico pump and a micromanipulator (WPI, Sarasota, FL, USA). After implantation with cancer cells, the ZF embryos (including non-implanted controls) were maintained at 34 °C as a compromise between the optimal temperature requirements for fish and mammalian cells [24]. Up to 400 implantations were manually achieved per h, with survival rates of *>*80% up to the 6th dpi. Fluorescent image acquisition was performed using a Leica MZ16FA stereo microscope (Leica Microsystems GmbH, Wetzlar, Germany). Separate images of the various segments of the ZF embryos were blended together to form a composite image using Adobe Photoshop CS6 software (Adobe Systems, Mountainview, CA, USA).

### 4.6. Quantitative Real-Time PCR (qPCR)

Total mRNA of the primary culture and cancer cell lines was isolated using TRIzol Reagent (Invitrogen, Carlsbad, CA, USA) following the manufacturer’s instructions. Five hundred nanograms of RNA were reverse-transcribed using the iScript cDNA Synthesis Kit (BioRad, Hercules, CA, USA). Real-Time PCR was performed on the 7500 Real-Time PCR System (Applied Biosystems, Foster City, CA, USA) using the TaqMan gene expression assay mix (Applied Biosystems). Amplification was performed in a final volume of 20 µL containing 2× Universal master Mix (Applied Biosystems), 2 µL of cDNA in a total volume of 20 µL. The following markers were analyzed: OPG, JAG1, CXCR4, RANK, IBSP, TFF1, SPARC, HPSE, CTGF, MMP-9, LOX and CDH1. The stably expressed endogenous β-actin and HPRT and were used as reference genes. The amount of transcripts was normalized to the endogenous reference genes and expressed as n-fold mRNA levels relative to a calibrator using a comparative threshold cycle (*C*_t_) value method (∆∆*C*_t_). RNA extracted from MCF7 cell line was used as calibrator. Gene expression analyses were graphed by heatmap using R software (https://www.R-project.org/).

## 5. Conclusions

Finally, we propose an original approach to study the metastatic process and cancer cell aggressiveness comprising the use of patient-derived primary cultures in the in vivo ZF model. The primary culture in the ZF showed behavior resembling that of the patient’s medical history but differing from that of the cancer cell line sharing the same hormonal receptor status and could thus be used to better understand drug sensitivity and to identify both prognostic markers and markers that are predictive of response to therapy. These results highlight the importance of using near-patient models in bone metastasis research and outline new methods for the clinical implementation of this in vivo model.

## Abbreviations

BM	bone metastasis
CDH1	cadherin-1
CHT	caudal hematopoietic tissues
CTGF	connective tissue growth factor
CXCR4	C-X-C chemokine receptor type 4
dpf	days post fertilization
HSCs	hematopoietic stem cells
HPSE	heparanase
HPRT	hypoxanthine phosphoribosyltransferase 1
IBSP	integrin binding sialoprotein
IL6	interleukin 6
LOX	lysyl oxidase
MMP-9	matrix metallopeptidase 9
JAG1	jagged1
OPG	osteoprotegerin
PBS	phosphate buffered saline
PDX	patient-derived material
PThrP	parathyroid hormone-related protein
RANK	receptor activator of nuclear factor κB
RANKL	receptor activator of nuclear factor κB ligand
TGFβ	transforming growth factor β
SPARC	secreted protein acidic and cysteine rich
TFF1	trefoil factor 1
*C*t	threshold cycle
VEGFR1	vascular endothelial growth factor receptor 1
ZF	Zebrafish
